# Expanded Polytetrafluoroethylene Membranes for Vascular Stent Coating: Manufacturing, Biomedical and Surgical Applications, Innovations and Case Reports

**DOI:** 10.3390/membranes13020240

**Published:** 2023-02-17

**Authors:** Roberta Cassano, Paolo Perri, Antonio Esposito, Francesco Intrieri, Roberta Sole, Federica Curcio, Sonia Trombino

**Affiliations:** 1 Department of Pharmacy, Health and Nutritional Science, University of Calabria, Arcavacata, 87036 Rende, Italy; 2 Complex Operating Unit Vascular and Endovascular Surgery, Annunziata Hospital, 1 Via Migliori, 87100 Cosenza, Italy

**Keywords:** PTFE, membrane, covered stent, vascular disease

## Abstract

Coated stents are defined as innovative stents surrounded by a thin polymer membrane based on polytetrafluoroethylene (PTFE)useful in the treatment of numerous vascular pathologies. Endovascular methodology involves the use of such devices to restore blood flow in small-, medium- and large-calibre arteries, both centrally and peripherally. These membranes cross the stent struts and act as a physical barrier to block the growth of intimal tissue in the lumen, preventing so-called intimal hyperplasia and late stent thrombosis. PTFE for vascular applications is known as expanded polytetrafluoroethylene (e-PTFE) and it can be rolled up to form a thin multilayer membrane expandable by 4 to 5 times its original diameter. This membrane plays an important role in initiating the restenotic process because wrapped graft stent could be used as the treatment option for trauma devices during emergency situations and to treat a number of pathological vascular disease. In this review, we will investigate the multidisciplinary techniques used for the production of e-PTFE membranes, the advantages and disadvantages of their use, the innovations and the results in biomedical and surgery field when used to cover graft stents.

## 1. Introduction

Expanded polytetrafluoroethylene (e-PTFE) is a hydrophobic polymer obtained by stretching PTFE, a fluoropolymer commonly used in the biomedical field for the manufacture of catheters for angioplasty, orbital implants, orthopedic joint implants, and membranes for stent coating [1]. Expansion occurs during the manufacturing process, when the solid material is modified into a porous lattice. The presence of negative charges on the polymer blocks the coagulation of blood proteins on the tissue surface and limits the activation of platelets [2].

e-PTFE is reasonably tough, and chemically inert; for this reason, in the biomedical field and in tissue engineering, this polymer has been used to make synthetic membranes capable of wrapping metal stentgrafts for the treatment of vascular diseases such as popliteal aneurysms, arterial perforations, iatrogenic perforations, and vessel stenosis [3]. The presence of a synthetic membrane, stretching between the stent struts and covering both the luminal and abluminal portions of the stent, is essential to create a physical barrier that can effectively prevent plaque protrusion, successfully sealing aneurysms, arterial perforations, or simply restoring blood flow in vessels prone to occlusion [4,5]. e-PTFE membranes have a porous and flexible structure and are chemically stable, biocompatible and inert, properties that enable them to resist degradation produced by microbiological or enzymatic reactions [6,7]. Fundamental characteristics of e-PTFE-membranes are non-thrombogenicity, the same viscoelasticity as native vessels and resistance to high blood pressure. In addition, when covered stent, they must be able to provide biocompatibility, accelerate the endothelization process, minimize vascular damage and reduce the proliferative response of the native artery. e-PTFE membranes are manufactured through stretching, spinning and pore-forming techniques [8]. Electrospinning can originate fibres with diameters ranging from a few nanometres to a few microns, presenting a high specific surface area and high porosity [9] that can be easily adjusted by changing the concentration of the spinning solution or the spinning parameters [10]. This characteristic also applies to the mechanical properties of the membrane [11]. e-PTFE membranes are often functionalized through covalent and non-covalent bonds, chemical impregnation, chemical surface modification, autologous vascularization, etc. These strategies are used to improve their properties and compatibility with blood tissue [12,13]. There are several commercially available e-PTFE covered stents for clinical use. The most commonly used ones consist of stainless steel (SS) metal frames with diameters ranging from 2. 5 to 16 mm with an e-PTFE film cover (iCAST, Atrium, Ekkatuthangal, Chennai, India), with an SS frame and struts embedded in an ePTFE film (Advanta V12, Atrium) [14], with an ePTFE film sandwiched between the SS matrix (JoStentGraftmaster Coronary Stent Graft or JoStent Peripheral Stent Graft, Abbott Vascular, Rome, Italy) [15] and with a Nitinol (NiTi) frame sandwiched between ePTFE (Symbiot Covered Stent, Boston Scientific, Pennsylvania Avenue, Washington, DC) [16]. There are also stents with a Nitinol frame covered with thin ePTFE films surrounding the metal frame (Viabahn Endoprosthesis, GORE, Bergheim, Austria) [17], or encapsulated with two thin ePTFE layers (Fluency + Vascular Stent Graft, BARD Peripheral Vascular, Becton, NJ, USA) [18]. The stents listed above are used in clinical conditions such as: treatment of tracheobronchial structures caused by the growth of cancerous tissue; treatment of iliac and renal arteries; treatment of aortic coarctation; treatment of aneurysms, fistulas, ruptures and perforations; and finally degenerated saphenous vein grafts [19]. The aim of this review was to provide an objective view of the production and manufacturing methods of e-PTFE membranes, their installation on vascular stents as a treatment option in the biomedical and surgical field (case report examples), their advantages and disadvantages compared to other polymeric membranes, and possible innovations in the literature regarding these devices.

## 2. e-PTFE Membrane Preparation

e-PTFE membrane preparation started mainly in the early 1960s. Today, various techniques including stretching, pore-forming, sintering, wrapping, electrospinning and near field electrospinningare used to produce them.

### 2.1. Stretching and Pore-Forming Process

**Figure 1:**
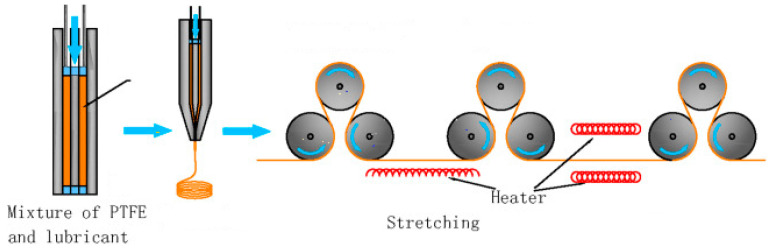
Stretching process.

A fine e-PTFE powder is homogeneously dispersed in an oil lubricant (processing aid) to become a paste. The first step, called pre-forming, typically takes place between 21 and 25 °C and serves to align the PTFE particles for packing in the best possible way. The presence of lubricants is essential to promote rapid absorption of the mixture [20]. The resulting paste is pressed into a mould and ram extruded into sheets or rods that are passed several times through two heated rolling mills to obtain a precise thickness. The resulting membranes are then stretched and hardened at room temperature and sintered at 340–365 °C, maintaining the stretching condition to fix the porous structure and prevent shrinkage [21] (Figure 1).

### 2.2. Sintering Process

In the sintering process, the polymer particles are only fully melted on the outer surface, so there are still many microchannels in the material that form a network of through holes [22]. As thetemperature, the surface of the attached particles melts further and firmly due to the movement and diffusion of the molecular chain segments, resulting in a porous film [23]. Sintering is a process that influences the final properties of the membrane as it improves its porosity usinginorganic agents (e.g., ZnAc_2_, NaCl and BaCl_2_) that are used as additives [24]. These additives are introduced and uniformly mixed with the polymeric materials, favouring their formation upon removal through post-processing or chemical attack [25].

### 2.3. Wrapping Process

The wrapping technique is used to make e-PTFE hollow-fibre membranes whose porosity increases as the stretching ratio increases. Membranes with large pore sizes are not suitable for use in various areas of technology or in the biomedical sector, as they do not guarantee adequate microfiltration [26]. Instead, by means of the wrapping method, it is possible to wrap ane-PTFE membrane with a small pore diameter on the outer surface of a PTFE hollow membrane with a large pore diameter to form a double layer that generates an asymmetric hollow membrane with 81% porosity and a pore size of 0.2 μm [27].

### 2.4. Near-Field Electrospinning Process

**Figure 2:**
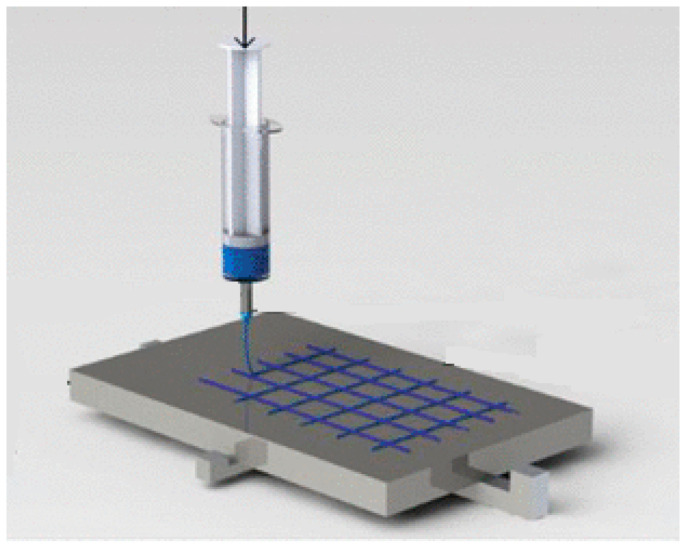
Near-field electrospinning process.

Near-field electrospinning (NFES) is a technique that allows membranes to be made by layering polymers on a planar substrate under the action of an electric field. Compared to conventional electrospinning technology, it favours a shorter spinning distance, a lower supply voltage, a more complex structure and controlled deposition [28] (Figure 2). The advantage of using this technique is the realization of membranes with a regular geometric pore structure [29]. In the biomedical field, electrospun fibrous scaffolds with adequate porosity, nanoscale topography and interconnectivity provide an ideal model for biomedical engineering as human tissues and/or cells are able to attach and organize themselves appropriately around fibres with diameters smaller than their own [30].

### 2.5. Electrospinning Process

Electrospinning is a technique that allows polymeric fibres with nano/submicron dimensions to be produced using an instrument consisting of an injector with a blunt tip, a booster pump to control the extrusion speed of a polymer solution, a DC electric field and a grounded collector [31]. In detail, a polymer fluid is able to pass through a high-voltage electric field generating a microjet that is solidified on a substrate as a fibre membrane layer [32]. In the case of e-PTFE membranes, it is necessary to add additives to the emulsion to be able to be produced by this technique because PTFE is very viscoelastic, and it is difficult to spin it into fibrils [33]. In an electrospun PTFE membrane, the pores are generated by the accumulation of nanofibrils, so it is easier to control their thickness, porosity, and diameter. To obtain a pure e-PTFE membrane, a sintering process is required to remove the additives after formation [34].

## 3. e-PTFE Membrane Used in the Design of Covered Stents

A covered stent consists of a thin synthetic e-PTFE membrane sleeve that may cover the inner lumen or abluminal surface (outer surface against the vessel wall) of the metal scaffold, or completely cover the stent in a sandwich-like configuration [35]. This thin membrane decreases the radial pressure of the stent and reduces the incidence of SRI and re-embolization by sealing the endoluminal layer with a physical barrier between the vessel wall and blood flow to limit tissue growth and prevent the release of thromboemboli [36]. Metal stents and e-PTFE membranes must be elastic enough to adequately expand to resist or overcome external forces from the vessel wall to avoid so-called metal scaffold recoil (a major cause of stent malposition and late thrombosis due to lack of coverage on the stent surface) [37]. For this reason, the total length of the stent should correspond to the length of the membrane [38]. The membranes covering the stent must withstand high pressures (>500 mm H), have low water permeability (to seal the perforated artery or prevent aneurysm growth), be hemocompatible (so as not to cause inflammatory or unwanted reactions), and be capable of undergoing surface modification to allow presentation of biomimetic peptides, antibodies and growth factors or to incorporate nanomaterials and therapeutic drugs for localized administration [39]. In this regard, stents covered with synthetic membranes can also serve as efficient drug delivery platforms, providing uniform coverage of the arterial wall and increased surface area for lesion coverage [40]. Furthermore, the presence of an e-PTFE coating on the surface of the stent can reduce the need for emergency surgery as its deployment time is relatively short (4 to 15 min), thus avoiding fluid spillage. The deposition of a layer of e-PTFE on a metallic stent can take place either by electrospinning, exploiting the electrostatic attraction between the needle of the syringe and the ground so as to generate an electrically charged jet of polymer solution in the form of nanofibres while the stent is rotated axially; or through immersion techniques in which the stent mounted on a rotating stainless steel or glass support is introduced into a mould filled with polymer and the polymer is poured over it at a controlled and predefined rate; or it may be through wrapping methods that consist of covering the outer surface of the stent with a previously prepared polymeric thin film, followed by suturing and bonding to the metal surface [41,42].

## 4. The Advantages and Disadvantages of e-PTFE Membranes as Stent Coatings

e-PTFE membranes used for the coating of metallic stents are relatively inert materials with a low friction coefficient and non-adhesion characteristics that provide durable and degradation-resistant properties [43]. They are highly biocompatible, and their inertness often prevents cells from adhering to the prosthesis, while their microporous structure prevents cell penetration and tissue growth [44]. However, under certain circumstances biological reactions such as adhesion of blood components (serum albumin, fibrin proteins, platelets) responsible for graft occlusion through activation of neointimal hyperplasia may occur on them [45]. For example, e-PTFE is 40–50% effective for large-diameter arterial grafts, while smaller calibre grafts only guarantee 20–25% permeable as it can cause thrombosis [46]. Today, one of the advantages of enhancing the properties of e-PTFE and making them favourable for coating vascular stents is their chemical functionalization through the formation of covalent and non-covalent bonds, with drugs and/or substances that can prevent the process of restenosis, bacterial infections, etc. [47]. In particular, chemical impregnation, chemical surface modification, autologous vascularisation and cell seeding can be promoted [48,49]. In order to give these membranes an antibacterial and anti-inflammatory action, it is possible to carry out a chemical reduction in the e-PTFE surface by UV-grafting polymerisation [50], a surface irradiation with gamma rays for immobilization of silver nanoparticles [51], a surface oxidation with grafting and covalent immobilisation of PLGA nanoparticles [52], and finally it is possible to subject them to an antibiotic pre-soaking [53]. To avoid the process of restenosis, endothelialization, platelet and/or cell adhesion typical of coated stents, e-PTFE membranes can be subjected to autologous vascularisation [54], spin-coating with polyurethane (PU) followed by deposition of PU nanoparticles [55], poly(1, 8-octanediol-co-citrate) spin-shearing method and covalent bonding of heparin (drug eluting stent) [56], seeding of autologous endothelial cells on the luminal surface [57], coating with a thin layer of thermoplastic styrene-ethylene propylene styrene copolymer (SEPS) [58] and/or grafting of the SEPS layer and covalent bonding with heparan-like semi-synthetic molecules sulphate (Patent EP 1501565 B1 [59].In this regard, it is important to emphasise the use of drug eluting stents, devices used in the prevention of restenosis that are capable of releasing bioactive agents into the blood stream that can be deposited in the tissues adjacent to the stent [60,61]. These drugs of an anti-inflammatory, immunomodulatory or anti-proliferative and antithrombotic nature (heparin, sirolimus, paclitaxel, etc.), may simply be bound to the surface of the stent, incorporated and released within polymeric materials (e-PTFE membranes) covering the stent (strutadherent), or they may cross (strut-spanning) the stent struts and/or be released by vectors [62]. In the case reviewed by Gohbara et al., good apposition of the e-PTFE-covered stent was confirmed in a patient with restenosis caused by DES. In particular, the possibility of stent thrombosis was found as the mechanical valve used was a VKA together with a P2Y12 inhibitor (prasugrel) [63].Compared to stents covered with materials such as PU, PTFEP (poly(bis (trifluoroethoxy) phosphazene), poly(ethylene-co-vinyl acetate) (PEVA), poly(n-butyl methacrylate) (PBMA), and poly(styrene block polymers -b-isobutylene-b-styrene) (SIBS) [64], those coated with e-PTFE possess the advantage of having better endothelialization depending on the pore size. The greater conformability of e-PTFE should allow the material to collapse easily which does not happen with other polymers whose stiffness may promote the possibility of kinking and disrupting flow [65]. They also possess a significantly smaller void than other grafts due to its inherent hydrophobic nature that produces a natural barrier to water that prevents blood permeation [66]. However, PU, PTFEP, PEVA, PBMA couplings have been developed to improve the low flexibility and poor adherence of ePTFE ones (as they require a high post-expansion pressure). Furthermore, they are biocompatible and relatively elastomeric, and have a low coefficient of friction and a non-stick surface [66].

## 5. Clinical Applications of Covered Stents

### 5.1. Popliteal Aneurysms

Dilatation of the popliteal artery is responsible for the formation of the popliteal artery aneurysm (PAA), an insidious pathology as it runs asymptomatically in most cases. The formation of an aneurysm causes the blood inside it to flow in a whirling manner and this causes the blood to stagnate at certain points in the aneurysm sac, which then becomes a clot and finally a thrombus [67]. The latter under the pressure of the cardiac systole can embolize and distally occlude the arteries that originate from the popliteal (tibial vessels) or even, if the thrombus is small, the arterioles of the foot with a phenomenon called ‘microembolization’ [68]. In these cases, the clinical onset is pain, pallor and hypothermia of the leg or foot because the embolus has produced a reduction in the vascularization of the limb with a consequent picture of ischaemia that will be more or less severe depending on the distal vessels involved [69,70]. The standard treatment for this pathology involves performing a surgical bypass to drain the haematoma and restore the blood supply to the foot. However, in the last ten years, there has been a move towards an endovascular approach that uses less invasive methods to restore blood supply by inserting a covered stent inside the popliteal artery itself [71]. Most cases reported in the literature refer to the use of the VIABAHN GORE, an e-PTFE membrane-coated stent that is superficially functionalized with heparin [72]. The positioning of this stent-graft allows a rapid switch from angiography to treatment, reducing the time of ischaemia of the lower limb. In the example reported by Oga et al., two clinical cases of patients complaining of coldness and pallor in the right lower extremity with suspected thrombus in a part of the popliteal artery, and one clinical case of a patient with posterior swelling of the left popliteal fossa due to haemorrhage in the artery itself were analysed. In all three cases, the implantation of a VIABAHN 5 × 50 mm stent (first case), a VIABAHN 8.0 × 100 mm stent (second case), and a VIABAHN 6.0 × 100 mm stent (third case) was performed. In the first patient, the postoperative follow up showed no ischaemic symptoms in the lower limb and ultrasound confirmed graft patency; in the second case, before confirming graft patency, an incision had to be made for release for compartment syndrome and the patient’s limb was severed.; finally, in the third case, a covered stent graft confirmed dilatation of the lesion and haemostasis by final follow-up [73]. In the work reported by Elliot et al., the use of an e-PTFE-covered stent would pose a lower risk of infection than surgical bypass or interposition grafting, especially in patients with immunosuppression where any surgical incision to evacuate the haematoma would increase the risk of infection. Indeed, the membranes lining stents are unlikely to become breeding grounds for bacterial cells. In this study, it was observed that the use of such a device in a 68-year-old, immunocompromised patient with a 6.9 cm ruptured right PAA not only reduced the haematoma but also reduced the infectious risks arising from the classic surgical approach [74]. The use of an e-PTFE-coated stentgraft for the acute rupture of a large PAA has also been tested in patients with Behçet’s disease (BD), where a traditional approach would increase the risk of infection and death. Behçet’s aneurysm is characterized by an intense infiltration of inflammatory cells, especially around the vasa vasorum, and severe medial destruction with loss of the elastic membrane. The weakness of the vessel wall due to the loss of arterial wall integrity may predispose to the formation of pseudoaneurysms and their rupture. This case report aimed to show the mid-term results (three-year follow-up) of a patient with a ruptured PAA, suffering from BD. The results showed correct positioning of the stentgraft without any endoleak with gradual recovery 5 days after surgery and surgical vacuation of the haematoma by means of an internal popliteal approach. Specific endovascular revascularization protocols exist in the literature for patients with acute limb ischaemia due to thromboembolic complications resulting from popliteal artery aneurysm (PAA) [75]. These protocols are based on a combination of vacuum-assisted thrombo-aspiration to improve tibiopedal outflow and covered stent grafting to exclude the PAA. The results showed that in the 17 patients treated (all with PAA dimensions between 37.4 ± 11.2 mm), the use of VIABAHN resulted in mortality and amputation rates at 30-day follow-up of 0% and 5.9%. Secondary patency was achieved in all cases (100%), with freedom from re-intervention between 80.4% and 54.8% and limb salvage of 88.2% after follow-up of 6, 12 and 24 months [76]. Other case reports are given in the Appendix A section.

### 5.2. Covered Stent in Iatrogenic Perforations

Iatrogenic perforations are defined as accidental injuries resulting from surgical procedures performed on or near a blood vessel. Specifically, this condition is considered an abnormal reaction in response to a physical or metabolic injury [77] (e.g., wound healing, trauma to the artery induced during revascularization procedures). In most cases, they are appreciable in diabetic patients undergoing dialysis where the insertion of special catheters can cause stenosis in the venous outflow of the upper limb with an arteriovenous (AV) graft or fistula. In these cases, it is important to intervene through urgent endovascular surgery to save the affected site and avoid the complications associated with haemodialysis catheters if thrombectomy of the fistula is unsuccessful [78]. An endovascular-covered stenthas emerged as the approach to treating symptomatic instead of open surgical options [79,80]. Dukkipati et al. described the case of a patient on haemodialysis for 6 months ineligible for the creation of a fistula due to the suboptimal size of the vein. They proceeded surgically by creating a right brachial AV graft. Eight months after placement of the AVG, a leak occurred with thrombization of the basilic vein and partial coagulation of the cephalic vein draining the flow of the graft. To ensure the patency of this iatrogenic fistula, a 7 mm × 5 cm coated stent (Viabahn) was placed at the point where the graft drained into the cephalic vein. Post-operative angiogram monitoring showed good flow from the graft to the cephalic vein [81]. The choice of using a covered stent for the closure of an AVF in the common femoral artery in a diabetic patient with a complex clinical history was considered by Cuong et al. as a viable alternative after open surgery. Specifically, a 6 mm × 57 mm covered stent (BeGraft BENTLEY INNOMED) was inserted from the common femoral artery through the fistula to the femoral bifurcation. Post-intentional follow-up clearly showed a closure of the AVF [82]. The formation of an iatrogenic arteriovenous fistula after endovenous laser treatment of the great saphenous vein is a rare complication that can occur during percutaneous endovascular interventions [83]. Tohmasi et al. presented a unique case of an iatrogenic superficial femoral artery-common femoral vein fistula with high flow, resulting in right heart failure and distal deep vein thrombosis in which they intervened by placing a covered arterial stent that closure of the arteriovenous fistula and restoration of arterial function [84].The formation of an iatrogenic arteriovenous fistula (AVF) around the proximal subclavian artery is also an extremely rare event that can hardly be treated in open surgery as a deep dissection around the fistula surrounded by dilated veins would be required [85,86]. In a study coded by Sato et al., the case of a 64-year-old man with AVF between right subclavian artery and the right vertebral vein due to an accidental puncture of the right subclavian artery. In this case, an endovascular repair with a 13 × 50 mm Viabahn covered stent was deemed [87,88].

### 5.3. Use of Covered Stent in e-PTFE in Leriche Syndrome Treatment

Leriche syndrome also called aortoiliac occlusive disease (AIOD) is defined as a chronic extensive occlusive disease affecting the distal abdominal aorta with involvement of the iliac arteries [89]. The extent and location of the atherosclerotic occlusions in relation to the arteries determine the classification of the disease. AIOD, when symptomatic, classically presents with a triad of claudication, impotence and absence of femoral pulsation. Claudication is defined as cramp-like leg pain reproducible with exercise [90]. The best treatment for such a disorder is an endovascular approach whose advantages include the reduction in perioperative mortality and the possibility of treating distal lesions during the same procedure with primary patency rates at 2–5 years of about 60%. Endovascular management is traditionally performed by bilateral iliac stenting, often referred to as ‘kissed iliac stenting’ [91]. ePTFE-coated stents are used for AIOD because they offer a potential advantage in preventing in-stent restenosis and reduce the risk of distal embolization in complex disease. The membranes lining these stents prevent macrophage exposure to atherosclerotic tissue, reducing the secretion of cytokines and growth factors, and directly blocking smooth muscle cell migration and neointimal tissue growth. The coated stent is placed in the severely calcified vessel and is then aggressively dilated with high-pressure balloons. Stents suitable for this type of procedure are Supera (Supera^TM^ Abbott Vascular), Viabhan and Fluencywhich provide ample radial pressure to prevent restenosis and reocclusion [92]. In a study conducted by Fujihara et al. on patients with complex aortoiliac disease and a background of highly calcified lesions (44%), in-stent restenosis (14.3%), and complex lesions (14.3%) or Leriche’s syndrome (8%), it was observed that the application of a covered stent showed benefits in terms of patency compared to traditional metal stents. Specifically, the investigation demonstrated the high clinical safety and efficacy of the GORE^®^ VIABAHN^®^ VBX covered stent (W.L. Gore & Associates, Flagstaff, AZ, USA) with a technical success rate of 92.6% in 214 of the 231 cases examined, and a primary patency rate of 93.4% and 95.3%.Depending on the extension of the disease, Leriche is categorized in Type I involving the distal aorta and the CFA, Type II, the lesions extending to the CFA, and Type III, the lesions extending to the SFA, as in our case [93]. In the study reported by Giusca et al. was reported a case of an 81-year-old male patient withcritical limb-threatening ischaemia (CLTI) due to aortoiliac occlusive which was subjected to endovascular treatment. After pre-dilatation, an 8.0–39 mm Viabahn balloon expandable coated stent (Gore VRViabahn VR Endoprothesis, Gore Medical, Bergheim, Austria) was implanted in the left CIA [94,95]. El Samadoni published a paper on the use of ePTFE-coated balloon-expandable stents to reconstruct the infra-renal aorta with or without simultaneous reconstruction of the aortic bifurcation. The choice of these ePTFE-expandable stents is due to the fact that most lesions of the infrarenal aorta or aorto-iliac vessels are difficult to treat with endovascular therapy, so there is an increased risk of intraprocedural complications, such as flow-limiting dissection, vessel perforation and/or distal embolization. In the study under review, a total of 15 stents with diameters (8–12 mm) and lengths (41–61 mm) were used to treat seven patients with isolated focal stenosis of the middle-lower aorta, focal stenosis near the bifurcation, chronic total aortobiiliac occlusion. Technical success and immediate clinical success were achieved in 100% of all of them as confirmed by palpability of distal pulses, improvement of ambulation with no pain at rest. This means that the use of covered stents is able to reduce the risk of morbidity and mortality [96]. An example of other case reports is given in the Appendix A section.

## 6. New Generation Stent Graft

In recent years, efforts have been made to develop new stent graft devices by focusing on smart or reactive materials designed to have one or more properties that can be transformed in a controlled manner by external stimuli such as stress, humidity, electric or magnetic fields, light, temperature, pH or chemical compounds [97]. In this regard, shape memory materials (SMM) have been identified that can be transformed and fixed in a temporary form, recovering their original shape when subjected to an appropriate stimulus [98]. Such polymers would offer greater recoverable deformation (up to 400%), lower density, easy processing and fabrication techniques, easier adjustment of material processing properties (e.g., transition temperature, biocompatibility, biodegradability, stiffness, functional gradient), programmability and controllability of recovery behaviour than those normally used [99,100]. According to literature data, fibrous membranes based on nanofibres of shape-memory polyurethane have recently been realized that can react to external stimuli to cope with complex in vivo alterations when used as a coating for endovascular stents. Instead, Small et al. developed a stent combining nitinol with a shape memory polymer to create a device capable of removing blood clots [101], while Maitland et al. developed a laser-activated shape memory foam for the treatment of aneurysm occlusion [102]. Medical devices with flexible or even dynamic structures could represent a new generation of vascular endoprostheses. Furthermore, medical implants with morphing characteristics could be created through 4D printing technology, an innovative technique already applied to tubular structures and blood vessels [103]. He et al. developed a 4D-printed tissue engineering material with excellent biocompatibility and a shape memory effect based on photoreticulated polycaprolactone (PCL). With this material they subsequently fabricateda small-diameter stent with a deformable shape memory loop for deployment and recovery size to support stenotic lesions [104]. In addition, a self-folding porous tubular structure was 4Dprintedwith encapsulated growth factors that were released in vivo by thermoregulation [105].

## 7. Conclusions

Membrane-coated stents made from ePTFE, fabricated by stretching, pore-forming, sintering, and electrospinning techniques, have proven useful in the treatment of a variety of vascular disorders. ePTFE membranes act as a physical barrier to block the growth of intimal tissue into the lumen, prevent so-called intimal hyperplasia and late stent thrombosis, and play an important role in preventing the restenotic process, especially, when chemically and physically functionalized. This is confirmed by the cases reported in the appendix which support those reported in the literature. In conclusion, the membranes described in this review can be used as a valid therapeutic option during emergency situations and to treat a number of pathological vascular diseases.

## Figures and Tables

**Figure A1:**
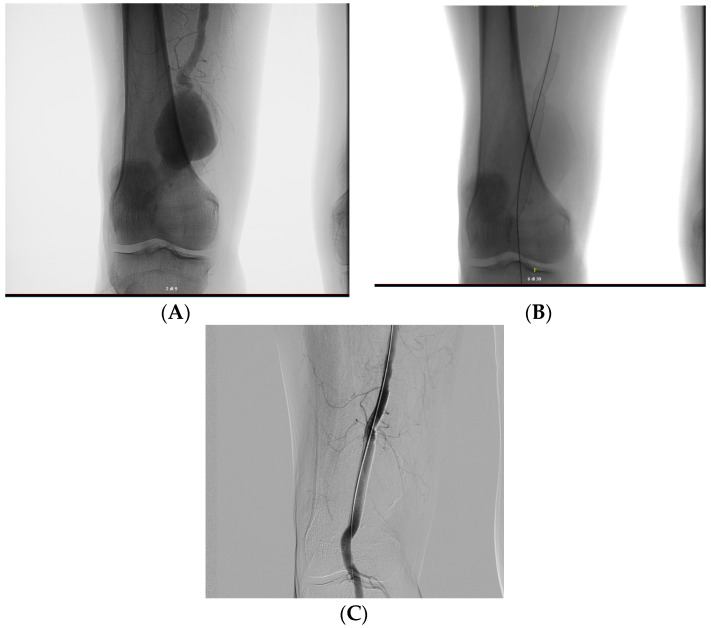
Angiogram of pre- (**A**), during (**C**) and post- (**B**) endovascular treatment.

**Figure A2:**
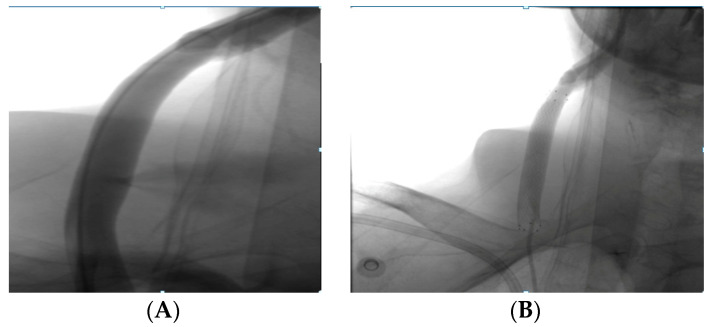
Intra-operative (**A**) and post-operative (**B**) angiogram.

**Figure A3:**
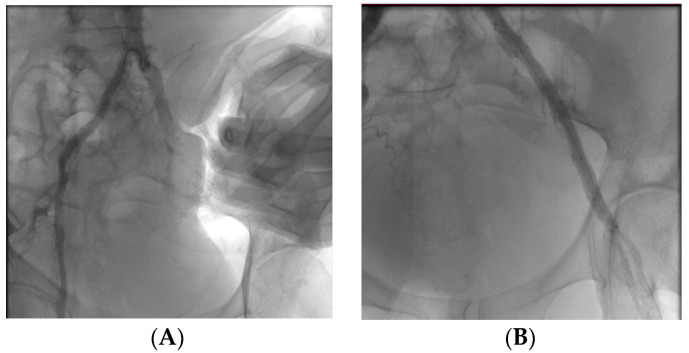
Multiple stenosi observed with angiography (**A**) and follow up post-operative (**B**).

## Data Availability

Not applicable.

## Appendix A

### Appendix A.1. Case Report: Popliteal Aneurysm

An 87-year-old patient with paroxysmal atrial fibrillation comes to the emergency department for coldness and pallor in the right lower extremity. He undergoes urgent ECD (echocolorDoppler) and low molecular weight heparin. The ECD reveals a dilation of the right popliteal artery, so a CT scan with contrast medium is requested, which shows the presence of a popliteal aneurysm with partial parietal thrombosis. The patient was a candidate for endovascular surgery via antegrade ultrasound access to the ipsilateral common femoral artery; intraoperative angiography was performed from the diagnostic catheter, which showed the presence of the popliteal aneurysm and the landing zones for proper stent release. After removal of the angiographic catheter, a VIABAHN 9.0 × 150 mm e-PTFE coated stent was released into the right popliteal artery with subsequent reshaping using a MUSTANG angioplasty balloon. Intra-operative and post-operative follow-up (ECD) revealed complete patency of the stent and absence of endoleak.

### Appendix A.2. Case Report: Bilateral Popliteal Aneurysm

A 79-year-old patient suffering from arterial hypertension and already undergoing right femoral-popliteal aneurysm THA/stenting in 2020 is admitted for surgery for a left popliteal aneurysm. Before undergoing surgical treatment, diagnostic examinations including ECD (echocolorDoppler) and CT scan with contrast medium were performed, which showed the simultaneous presence of both the popliteal aneurysm and an abdominal aneurysm. This confirms the data in the literature which state that in 20% of cases it is possible to make an incidental diagnosis of an abdominal aneurysm in patients with a right or left popliteal aneurysm. The patient was a candidate for endovascular surgery through antegrade ultrasound access on the right common femoral artery; intraoperative angiography was performed from the diagnostic catheter, which showed the presence of the popliteal aneurysm at the inferior III of the left femoral artery in its proximal suprageniculate tract and the landing zones for the correct release of the stent. After removal of the angiographic catheter, a VIABAHN 7.0 × 120 mm e-PTFE coated stent was released with subsequent reshaping of the stent using a MUSTANG angioplasty balloon. Intraoperative and post-operative follow-up (ECD) revealed complete patency of the stent and absence of endogen from upstream to downstream.

### Appendix A.3. Case Report: Iatrogen Perforation

A 78-year-old patient is admitted to nephrology for dialysis. While attempting to place a CVC for haemodialysis in the right laterocervical region, there is a pulsating swelling on the neck. On ECD a pseudoaneurysm of about 4 cm is detected, fed by a small breach on the common carotid artery in the lateral region. A CT angiogram was performed, which confirmed the presence of the pseudoaneurysm at the middle III of the common carotid artery with a leak of contrast medium from the same at the site of a previous accidental puncture. Verifying the risk of suffocation due to accumulation of blood at the base of the neck, an urgent surgical intervention is performed by placing a COVERA PLUS stent (9 × 60 mm) to block the breach in the carotid artery. Intra-operative and post-operative angiographic control is performed to detect the perviousness of the vessels downstream of the stent and downstream of the right carotid bifurcation. After the endovascular treatment, a surgical cut is made in the vicinity of the haematoma created to allow the blood to flow out and the pouch to empty.

### Appendix A.4. Case Report: Leriche Syndrome

A 69-year-old patient with hypertension, BPCO and diabetes mellitus comes to the outpatient clinic complaining of free walking intervals of less than 50 m on flat ground, with associated cramping pain in the thigh and calf. Control ECD is performed which shows demodulated flow downstream of the iliac artery with velocimetry less than 30 cm/sec systolic peak. A control CT is requested which confirms a Leriche syndrome characterised by bilateral iliac stenosis with increased calibre of the lumbar arteries, left and right iliac stenosis and aortic stenosis. From a surgical point of view, we opted for ultrasound-guided puncture on the left side with placement of the 7 fr introducer on the right and left side in the common femoral artery. Angiography was performed with evidence of multiple stenosis on the iliac and external bilaterally involving the aortic carrefour. Viabahn8 × 50 mm stents were placed on the right and s common iliac and 7 × 100 mm and 7 × 50 mm Viabahn stents were placed on the right and s external iliac, respectively. Remodelling was performed on 7 × 80 and 6 × 60 Mustang flask. The final check showed perveity of the right hypogastric.
